# Research on the Mechanism of Influence of Game Competition Mode on Online Learning Performance

**DOI:** 10.3390/bs12070225

**Published:** 2022-07-10

**Authors:** Hailing Xu, Shilun Ge, Feng Yuan

**Affiliations:** 1 Academic Affairs Office, Jiangsu University of Science and Technology, Zhenjiang 212003, China; 2 School of Economics and Management, Jiangsu University of Science and Technology, Zhenjiang 212003, China; jzgsl@jzerp.com; 3 Graduate School, Jiangsu University of Science and Technology, Zhenjiang 212003, China; yfjuster1979@163.com

**Keywords:** online learning, learning performance, competition, gamification, curriculum platform

## Abstract

With the rapid development of information technology and the influence of the COVID-19 pandemic, online learning has become an important supplement to the teaching organization form of basic education and higher education. In order to increase user stickiness and improve learning performance, gamification elements are widely introduced into online learning situations. However, scholars have drawn different conclusions on the impact of game-based competition on online learning performance. This study is based on field theory and constructivist learning theory. Taking the online interaction of the curriculum platform as the situation, psychological capital as the intermediary variable and connected classroom atmosphere as the adjustment variable, this paper constructs an interaction model between game competition and online learning performance and discusses in depth the intermediary effect of psychological capital and the adjustment effect of a connected classroom atmosphere. The results show that game-based competition has a significant positive effect on learning performance, and the effect of direct competition is better than that of indirect competition; the self-efficacy dimension of psychological capital plays an intermediary role between direct competition and learning performance, and the resilience dimension plays an intermediary role between competition and learning performance; and a connected classroom atmosphere plays a regulating role in the dimensions of game competition, knowledge mastery and knowledge innovation.

## 1. Introduction

According to the 47th Statistical Report on Internet Development in China released in 2021, as of December 2020, the number of online education users reached 342 million [1]. Only the online learning platform of the massive open online national excellence courses (icourse163.org accessed on 1 May 2022) of Chinese universities has been downloaded and installed (300 million times) in the Huawei App Store. During the period of the COVID-19 pandemic, universities and primary and secondary schools in many provinces adopted online teaching to conduct teaching activities. With the rapid development of information technology and the influence of the COVID-19 pandemic, online learning platforms have become a universal medium for knowledge acquisition and online teaching.

With the deepening integration of online learning and education represented by MOOC, scholars have begun to pay attention to the learning performance of online learning [2]. Scholars hope to improve the effect of online learning through the design of the online teaching mode, the application of teaching evaluation, teachers’ teaching competence, learning participation and learning willingness [3,4,5]. In the past two years, with the popularity of the concept of gamification, online platforms have introduced the elements of gamification competition into the online learning scene to better stimulate the learning interest, learning experience and learning effect. Scholars have found significant differences in the effect of game competition on online learning performance. Specifically, Burguillo, Cagiltay and Chen Guoqing found that the game-based competitive environment is conducive to promoting learners’ learning motivation, and to improving learning frequency and academic performance. Fox, Harms and Christy found that game competition, such as rankings, reduces learners’ academic performance. The reasons for this result may be as follows: on the one hand, existing analysis of the role of gamification competition and learning performance rarely classifies the gamification competition mode in the online learning context; on the other hand, the game-based competitive situation of online learning is mainly set in an interactive learning link, which is manifested as competitive activities represented by rankings and competitions, and the learners’ own characteristic variables and atmosphere factors in competitive learning activities are the influencing variables that cannot be ignored. Based on this, this paper classifies the game-based competition modes in the existing online learning situations, explores the influence of game-based competition on online learning performance, and puts forward the intermediary role of psychological capital, which describes the characteristic variables of individual states, and the moderating effect of a connected classroom atmosphere, which describes environmental factors.

## 2. Connotation of Core Variables

Gamification is defined as the use of gamification elements in a non-gamification environment [6,7], such as game thinking and game mechanisms. Gamification is based on game mechanics, esthetics and reasoning to motivate and promote learning [8], and the main difference between ludification and gamification is that the former prioritizes the recreational aspect [9], whereas the latter matches the educational curriculum with skills students will need in their lives [10], focusing on competences. Based on this study, gamification is described as introducing the competition mechanism of games into online learning situations and learning curriculum knowledge through competitive activities.

### 2.1. Game-Based Competition in the Online Learning Context

#### 2.1.1. Game-Based Competition Relationship in the Online Learning Context

The competition of learning subjects participating in the learning process based on online learning platforms mainly focuses on teacher–student interaction, student–student interaction and the game-based learning process. Unlike static video learning, in the process of teacher–student interaction or student–student interaction, there is a competitive relationship among online learners. In order to obtain excellent performance and gain recognition from teachers and other learners, or more points, learners participate in the competition through online discussion, expressing opinions, answering questions, designing work online, etc. In the game-based learning interaction, learners complete the learning content according to the learning rules, obtain learning points, reach the corresponding learning level or point ranking, receive honors or dividends and form an indirect competitive relationship; in online competition or group competition, learners challenge each other in a virtual environment, confront each other directly and form a direct competitive relationship.

#### 2.1.2. Game Competition Mode in Online Learning Situation

The competitive relationship of online learning is embodied in direct competition and indirect competition of face-to-face confrontation. Under different competitive relationships, the competition modes designed by knowledge platforms and knowledge exporters for learners mainly include the competition mode, ranking mode and grade competition mode.

##### Direct Competition: The Competition Mode of Competition

In the competition mode of competition, individuals compete through one-to-one direct confrontation, where the winning party in the competition will compete successfully. Confrontation can be individual confrontation or group confrontation, such as double confrontation, challenge and four-person challenge. The core of the competition is that at least two players or two groups of players compete for advantages around goals and tasks [11], which is the driving force for promoting learners’ participation in learning activities and improving their input in online learning situations. In online learning situations, in a one-to-one competition, teachers give learning tasks, and individuals (or groups) participating in the competition learn and answer the learning tasks, thus completing the learning tasks. The winning individuals (or groups) obtain corresponding points or scores; in multi-person (or group) competition, aiming at the learning tasks given by teachers, the individuals (or groups) participating in the competition participate in the learning and completion of the learning tasks at the same time and obtain corresponding points or scores according to the completion time and accuracy. In the competition mode of direct competition, learners participate in the competition of learning under the condition of understanding the competition system, gain self-affirmation and pride of winning through the competition system and, at the same time, understand the shortcomings from failures, so as to constantly improve the blind spots of knowledge.

##### Indirect Competition: Ranking Mode

In the ranking competition mode, the performance of participants participating in activities or completing tasks is evaluated, and the evaluation results are sorted. Rankings can compare the performance of a large number of participants by ranking their achievements in tasks. The ranking list is open to the whole competitive group, and the ranking level in the ranking is the recognition of the knowledge ability of the participants in a certain stage and specific knowledge field. In a sense, it is a symbol of the status of the participants in this stage. Anderson and other scholars believe that the status in the peer group has an inspiring effect. Even if the symbols symbolizing this status are insignificant, people still pay great attention to them and make active efforts to gain recognition [12]. In the online learning situation, indirect competition promotes or inhibits participants’ online learning behavior by comparing their learning results. Participants invest more time and energy in consolidating and expanding their knowledge reserves in order to achieve a higher ranking, thus showing some expected behaviors.

##### Indirect Competition: Hierarchical Competition Mode

In the hierarchical competition mode, competition is based on dividing grades. Participants of the same grade gain the right to advance to a higher level through competition, while participants who fail in competition lose the opportunity to advance to a higher level. The biggest core advantage of this mode is that participants can compete at the same level, and it constantly allows participants to experience the joy of victory, so as to gain self-confidence and motivation to continue. In the online learning situation, teachers divide learning tasks or interactive topics into different difficulty levels. Online learners complete learning tasks from the basic level, and learners who successfully pass customs can be promoted to a higher level; otherwise, they will continue to learn and accumulate at the original level. The hierarchical competition mode divides learners into knowledge difficulty levels and helps learners set stage learning goals. When they complete the learning goals and tasks of the current level, they will be promoted to a higher level and adjust new learning goals, so as to realize the continuous accumulation of knowledge and the continuous improvement of ability.

### 2.2. Online Learning Performance

Online learning performance is the core index to evaluate and test the quality of online education and teaching [13]. In 2004, the American Association of Educational Communication and Technology defined learning performance as the ability of learners to use newly acquired knowledge and skills, which not only refers to the acquired basic knowledge and skills, but also the ability to use them flexibly [13]. Learning performance refers to the results of learning, and the understanding and mastery of knowledge. According to different dimensions, there are different classifications of learning performance. Many researchers divide learning performance into short-term learning performance, medium-term learning performance and long-term learning performance according to the time dimension [13]. Some researchers divide learning performance into the retention effect and transfer performance according to the degree of knowledge mastery [14]. Kirkpatrick put forward a theoretical four-level evaluation model of learning performance in “Techniques for Evaluating Training Programs”, including four levels: reaction, learning, behavior and result [15,16].

Online video learning is the main link of online learning. Online video learning performance is the accumulation of knowledge cognition, processing, application and innovation in the time-series and evaluation dimensions after watching, understanding and digesting video content. Learners first have a preliminary cognition and understanding of the knowledge content through online video learning in the cognitive memory layer and then enter the understanding processing layer. By absorbing and digesting knowledge, they can draw inferences from others, apply knowledge to practice and reach the knowledge transfer application layer. Then, they can realize knowledge innovation through knowledge integration and upgrading. In the learning process, knowledge not only circulates internally in the cognitive memory layer, understanding processing layer, migration application layer and innovation promotion layer, but also externally among the four layers to improve learning performance. In this study, online video learning performance is measured from three dimensions: knowledge mastery, knowledge application and knowledge innovation.

## 3. Hypothesis Study

### 3.1. The Relationship between Competition and Learning Performance

Due to the popularity of the game-based competition concept in various fields, the concepts of direct competition and indirect competition presented by the competition mode, ranking competition mode and hierarchical competition mode have been widely introduced into learning situations in an attempt to improve learners’ learning motivation and learning performance [17,18,19]. However, Harms and Fox believe that game elements are widely used in teaching as a way to improve students’ participation in class. However, in a 16-week experiment of studying the same course, it was found that the students who adopted the game-based courses of rankings and badges showed lower motivation and lower final exam scores than the students who did not play games [20]. Christy and Fox found in a learning experiment of a virtual classroom that the competition of the ranking mode aroused the social comparison of female learners, affected their academic identity and academic performance and significantly reduced their mathematics test scores [21]. Burguillo conducted experimental research on students’ learning activities in a ten-year research cycle, based on friendship competition games, and implemented the framework of competition-based learning (CnBL) to stimulate students’ learning motivation and improve students’ academic performance. The results showed that the competition mode of friendly matches was helpful in improving students’ learning motivation and performance [22]. Cagiltay et al. took 142 students’ learning performance as the research object, introduced competitive game design elements and examined the effect of competitive elements on learning performance. The results showed that creating a competitive environment in serious games significantly improved learners’ learning motivation and academic performance [23]. Chen Guoqing and others took online language learning as the situation, from the perspective of “demand-availability-functional characteristics”, to stimulate the psychological occupation and self-satisfaction of individual learning through platform functional characteristics and availability [11]. The frequency of individual learning behaviors participating in direct competition and indirect competition in online language learning was significantly improved, and the weekly platform used stickiness 5.31 times more; the weekly learning performance (memorizing words) of learners participating in the direct competition group increased by 41%, while that of learners participating in the indirect competition group increased by 67.8%.

**Figure 1:**
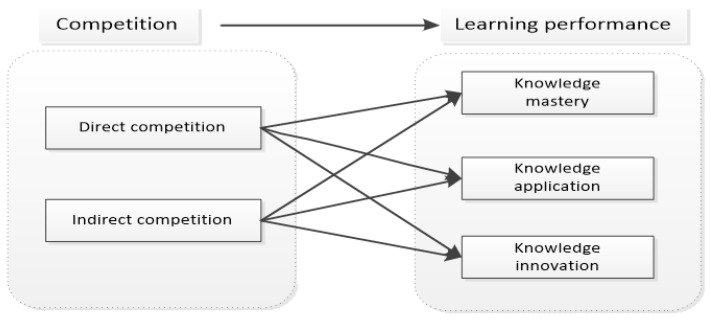
Mechanism model of competition’s impact on learning performance.

Existing studies introduced game-based competition into learning situations and explored the effects of competitive learning methods such as rankings, badges and friendly matches on learning performance, but there are still differences. According to Lewin’s field theory, people’s behavior depends on their living space. When individuals have internal needs, they form a psychological tension system, which leads to an introduction and rejection value and induces the generation of behavior. When competition triggers online learners’ competitive spirit, they can achieve a good performance and pleasant feeling in the process of competition and realize academic self-confidence, which is conducive to enhancing learners’ internal needs, enhancing the tension of individuals’ psychological tension system and improving the value of citation and rejection. In the online learning situation, individuals are also in a learning tension system. Competition, as the tension of the tension system that induces internal demand, triggers or hinders learners’ learning motivation, learning participation and engagement, and affects learning performance. Based on the theoretical inquiry and previous research, the research hypothesis model is put forward as shown in Figure 1:

**H1:**  *Competition has a significant impact on online learning performance.*

### 3.2. The Mediating Role of Psychological Capital

Luthans, an organizational behaviorist, thinks that psychological capital is a psychological state that can promote positive behaviors in the process of individual work and growth. Avey et al.’s meta-analysis of 51 analysis samples involving 12,567 employees found that psychological capital has significant effects on organizational commitment, job performance, job satisfaction, turnover intention, cynicism, job stress and anxiety [24]. Gu Jianghong and other scholars believe that employees or teachers with positive psychological states will invest more time and energy in their work, which is reflected in the difference in job performance [25]. For particular groups of college students, in addition to psychological capital reflecting the deep psychological state of the learners, the optimism, hope, resilience and self-efficacy of psychological capital are reflected in the whole learning process and have a direct impact on academic achievements [26]. Zhang Hongru analyzed 287 sample data of knowledge workers and found that the innovation process, innovation result and innovation performance of psychological capital have significant effects [27].

**Figure 2:**
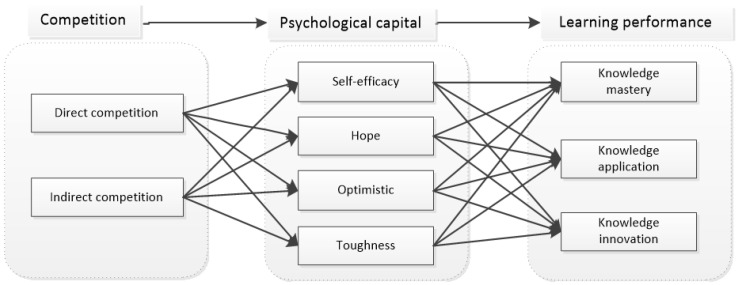
Mediation effect model of psychological capital.

According to Lewin’s field theory, individual behavior B is a function of the behavior subject p and psychological environment E, and the behavior subject is one of the decisive factors of individual behavior. Psychological capital is an individual’s characteristic and state variable, and it is an important factor affecting individual behavior. Therefore, specific to the online learning research situation in this paper, individuals with high psychological capital often regard competition as the exploration and strengthening process of learning objectives, regard competition as the driving force to maintain their continuous learning, regard the result of competition as a manifestation of learning achievements, enjoy the competition process, experience the fun of competition and enhance their self-confidence in learning. However, individuals with low psychological capital will regard competition as a type of pressure, which will cause anxiety, destroy the internal demand of learning, strengthen the negative impact of competition, reduce self-efficacy and even produce bad behaviors such as dropping out of class and experiencing weariness in learning. Based on the above analysis, the research hypothesis model is put forward as shown in Figure 2:

**H2:**  *Psychological capital plays an intermediary role between competition and online learning performance.*

### 3.3. The Adjusting Function of Connected Classroom Atmosphere

Li Xiuzhuang pointed out that classroom atmosphere has a guiding function, cohesive function, driving function, edifying function and discipline literacy construction function [28]. Classroom atmosphere plays a guiding role in learners’ values and codes of conduct and can guide learners’ codes of conduct to develop in the direction expected by society. Classroom atmosphere can also bring learning from different backgrounds together through classroom charm and internalize the sense of identity and belonging of learning. Moreover, classroom atmosphere drives learners to have autonomous learning willingness and behavior by meeting learners’ learning and communication needs. It can cultivate learners’ sentiment, and a good learning atmosphere helps learners to construct the meaning of professional knowledge and form discipline accomplishment [28]. Sidelinger and Booth-Butterfield found that the connectivity between learners can play a moderating role between teachers’ recognition behavior and students’ learning participation, and the connectivity of the classroom atmosphere has a significant impact on students’ sense of participation [29]. Even if teachers make teaching mistakes, students can still achieve positive learning results with the help of a connected classroom atmosphere [30].

**Figure 3:**
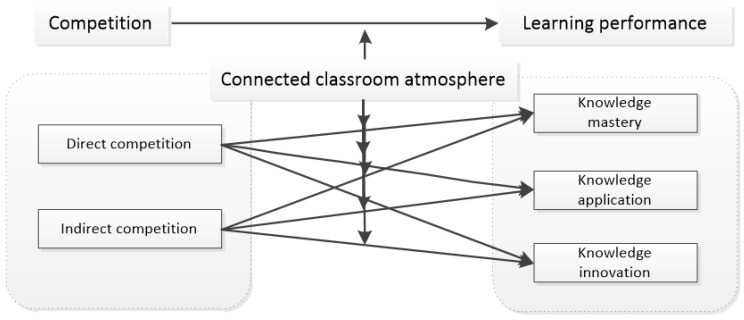
Modulation effect model of a connected classroom atmosphere.

According to field theory, individual behavior B is a function of the behavior subject p and psychological environment E, and the psychological environment is one of the decisive factors of individual behavior. Competitive interactive learning based on online virtual environments means that learners learn in interactive ways such as discussion, competition and answer-first in the discussion area, thus forming a connected learning classroom in online virtual environments. When learning in an online virtual learning classroom, a friendly classroom atmosphere helps learners to relieve the psychological pressure and anxiety brought by competition, relax and look at the learning results of competition and interaction with an optimistic attitude; on the other hand, a friendly classroom atmosphere can weaken learners’ concerns about facing problems caused by the retention of “traces of wrong answers” and actively participating in learning interaction. Based on the above analysis, the research hypothesis model is put forward as shown in Figure 3:

**H3:**  *A connected classroom atmosphere positively regulates the relationship between competition and online learning performance.*

## 4. Research Design

### 4.1. Data Collection

In order to improve the quality of the samples and ensure their representativeness, the random principle was adopted in this questionnaire. First of all, in the investigation area, representative education provinces (such as Beijing, Jiangsu, Zhejiang and Shandong) were selected to conduct the investigation and research work. Secondly, in the survey object, college students were selected as the core group of investigation and study, and questionnaires were randomly distributed. Thirdly, the survey showed that this questionnaire survey was purely academic research and was filled out anonymously. Additionally, all subjects provided their informed consent for inclusion before they participated in the study. Finally, 1200 questionnaires were sent out and collected, and 132 invalid questionnaires were eliminated (the elimination principle is the same as that in the small sample test), and 1068 valid questionnaires were obtained, with an effective questionnaire recovery rate of 89%.

### 4.2. Variable Measurement and Homology Test

Mental capital and classroom atmosphere were the mediators involved in this research model; existing mature measurement scales with high reliability and validity were adopted; and education experts, psychologists and teachers were invited to fine-tune the expression of the measurement terms. The intermediary variable of psychological capital refers to the measurement scales of Luthans (2007), Xu Haiyuan (2016) and Zhao Ruiyue (2021), with a total of 24 items. The regulating variable connected classroom atmosphere mainly refers to the measurement scales of Dwyer (2004) and Gong Yao (2019), with a total of 18 items. This study developed the measurement scales of the independent variable competition and dependent variable online learning performance involved in the research context. There are 5 items in the direct competition dimension measurement and 5 items in the indirect competition dimension measurement in the competition measurement scale, totaling 10 items. There are 5 items in the knowledge mastery dimension measurement, 5 items in the knowledge application dimension measurement and 5 items in the knowledge application dimension measurement in the learning performance scale, totaling 15 items.

We used the corrected item-total correlation to analyze and purify the measurement clauses. The corrected item-total correlation indicates the internal consistency of other items of the measurement item. The higher the coefficient value, the higher the internal consistency between the item and other items, and the lower the coefficient value, the lower the internal consistency between the item and other items. According to the test standard, when the CITC is less than 0.3, the measurement clause is deleted. When the CITC is between 0.3 and 0.5, if the reliability coefficient (Cronbach’s α) increases after deletion, the measurement clause will be deleted. Cronbach’s α was used to analyze the reliability of the measurement terms of each variable. The value of Cronbach’s α is between 0 and 1, and the larger the value of Cronbach’s α, the higher the internal consistency of the measurement terms, that is, the higher the reliability. In this study, the value of 0.7 proposed by Nunnally and Peterson was used as the reliability test standard. After deleting the direct competition measurement clause “I am willing to watch other people’s online learning competition activities”, the overall α increased from 0.819 to 0.838, so it was deleted. After deleting the indirect competition measurement clause “When I ask others for advice, they will impart knowledge or skills to me”, the overall α rose from 0.742 to 0.782, so it was deleted. After deleting the knowledge application measurement clause “I feel dizzy when I see the application questions”, the overall α rose from 0.804 to 0.865, so it was deleted. The final measurement scale was obtained after the evolution of the measurement terms. According to the homologous variance bias test of the sample data, 12 factors with features greater than 1 were extracted by a single-factor test, which explained 42.7% of the variance, and the maximum factor explained 18.69% of the variance, which was lower than 50%, indicating that there was no homologous bias problem in this study.

### 4.3. Reliability and Validity Test

The independent variable competition (C) includes two dimensions: direct competition (DC) and indirect competition (IC). The regulatory variable psychological capital (PC) includes four dimensions: self-efficacy (SE), hope (HO), optimism (OP) and resilience (TO). The adjustment variable is the classroom atmosphere (CA). The dependent variable learning performance (LP) includes three dimensions: knowledge mastery (KM), knowledge application (KA) and knowledge innovation (IA).

Cronbach’s α was used to test the reliability of the scale with four variables. The α value of direct competition was 0.840, the α value of indirect competition was 0.811 and the overall reliability of the competition scale was 0.889, indicating that the independent variable competition has good reliability. The alpha value of self-efficacy was 0.915, the alpha value of hope was 0.927, the alpha value of optimism was 0.925, the alpha value of resilience was 0.916 and the overall reliability of mental capital was 0.958, which shows that the intermediary variable mental capital has good reliability. The reliability of connected classroom atmosphere was 0.960, which shows that the moderating variables have good reliability. The alpha value of knowledge mastery was 0.820, the alpha value of knowledge application was 0.869, the alpha value of knowledge innovation was 0.839 and the overall reliability of learning performance was 0.902, which shows that the dependent variable learning performance has good reliability.

The scale is divided into four dimensions: competition, psychological capital, interconnected classroom atmosphere and learning performance. Competition is a two-dimensional construct, psychological capital is a four-dimensional construct and learning performance is a three-dimensional construct. Therefore, it is necessary to test the construct validity of three scales: competition, psychological capital and learning performance. Exploratory factor analysis was used to test the validity of the scale. The KMO value of the competition scale was 0.850, which is greater than 0.8, meaning it has a good standard. At the same time, the chi-square value of the Bartlett spherical test was 954.852, and the significance level was *p* = 0.000, which shows that there are common factors among the measurement clauses of competition and that it is suitable for factor analysis. There were two factors whose characteristics of competition were greater than 1. After the main axis factor extraction, the cumulative interpretation variance of the two factors was 72.069%, which indicates that the competition scale has good construct validity. The KMO value of the learning performance scale was 0.931, which is greater than 0.8; the chi-square value of the Bartlett spherical test was 2491.825 and the significance level was *p* = 0.000, which shows that there are common factors among the measurement clauses of learning performance and that it is suitable for factor analysis. There were three factors whose characteristics of learning performance were greater than 1, and the cumulative variance of the three factors on the orthogonal rotation axis after extraction was 84.795%, which shows that the learning performance scale has good construct validity. The KMO value of the psychological capital scale was 0.959, which is greater than 0.8, which means it has a good standard. At the same time, the chi-square value of the Bartlett spherical test was 6015.362, and the significance level was *p* = 0.000, which shows that there are common factors among the measurement clauses of psychological capital and that it is suitable for factor analysis. There were four factors whose characteristics of psychological capital were greater than 1, and the cumulative variance of the four factors on the orthogonal rotation axis after extraction was 87.941%, which shows that the psychological capital scale has good construct validity.

## 5. Hypothesis Testing

### 5.1. Influence Analysis of Control Variables

An independent sample T-test and one-way ANOVA were used to test the effects of control variables on mental capital, classroom atmosphere and learning performance. The results of the independent sample T-test showed that gender had no significant difference in mental capital, classroom atmosphere and learning performance with 95% confidence. The results of the one-way ANOVA showed that the influence of place of origin, education level, grade, degree category and MOOC learning experience on mental capital, classroom atmosphere and learning performance had no significant difference at the 95% confidence level.

### 5.2. Test of the Effect of Independent Variables

Multiple regression analysis was used to verify the relationship between competition and learning performance. The minimum tolerance of the explanatory variables of the regression model was 0.671, and the maximum value of the variance expansion factor was 1.665, which indicates that there is no collinearity among the variables.

From Table 1, we can see that the F value of the regression model of knowledge mastery was 10.775, which was significant at the level of *p* < 0.05; the F values of the regression models of knowledge application and knowledge innovation were 12.911 and 11.403, respectively, which were significant at the level of *p* < 0.01. The regression coefficients of the dependent variable’s dimensions in the direct competition dimension of the independent variable competition were 0.237, 0.264 and 0.262, respectively, which were significant at the level of *p* < 0.01, indicating that the direct competition dimension of competition has a significant positive impact on knowledge mastery, knowledge application and knowledge innovation. The regression coefficients of the indirect competition dimension in knowledge mastery and knowledge application were 0.194 and 0.201, respectively, which were significant at the level of *p* < 0.01. It can be said that the indirect competition dimension has a significant positive effect on knowledge mastery and knowledge application. The regression coefficient of the indirect competition dimension in knowledge innovation was 0.158, which was significant at the level of *p* < 0.05, indicating that indirect competition has a significant impact on knowledge innovation. From the comparison of the regression coefficients, the regression coefficients of the direct competition dimension in knowledge mastery, knowledge application and knowledge innovation were greater than those of the indirect competition dimension, which shows that the positive effect of direct competition on knowledge mastery, knowledge application and knowledge innovation is higher than that of indirect competition.

### 5.3. Mediation Effect Test

#### 5.3.1. Correlation Analysis between Competition and Psychological Capital

Multiple regression analysis was used to verify the relationship between competition and psychological capital. The minimum tolerance of the explanatory variables of the regression model was 0.671, and the maximum VIF was 1.665, which indicates that there is no collinearity among the variables. The F values of the regression models of self-efficacy, hope, optimism and resilience were 19.698, 19.414, 13.486 and 14.642, respectively, which were significant at the level of *p* < 0.01. The regression coefficients of self-efficacy and resilience in the independent variable direct competition were 0.292 and 0.214, respectively, and were significant at the level of *p* < 0.01. The regression coefficients of hope and optimism in direct competition were 0.194 and 0.218, respectively, and were significant at the level of *p* < 0.05. This shows that direct competition has a significant positive impact on self-efficacy, hope, optimism and resilience of psychological capital. The regression coefficients of self-efficacy, hope, optimism and resilience in indirect competition were 0.320, 0.407, 0.348 and 0.344, respectively, which were significant at the level of *p* < 0.01, indicating that indirect competition has a significant positive influence on self-efficacy, hope, optimism and resilience of psychological capital. From the comparison of the regression coefficients, the regression coefficients of indirect competition in the four dimensions of psychological capital were greater than those of direct competition, which shows that the positive effect of indirect competition on psychological capital is higher than that of direct competition.

#### 5.3.2. Correlation Analysis between Psychological Capital and Learning Performance

Using multiple regression analysis, this paper verifies the relationship between psychological capital and learning performance in different dimensions. The minimum tolerance of the explanatory variables of the regression model was 0.348, and the maximum VIF was 2.575, which indicates that there is no collinearity among the variables. The F value of the knowledge mastery regression model was 25.502, which was significant at the level of *p* < 0.05; the F values of the regression models of knowledge application and knowledge innovation were 24.368 and 23.439, respectively, which were significant at the level of *p* < 0.01. The regression coefficients of knowledge mastery, knowledge application and knowledge innovation in the self-efficacy dimension of psychological capital were 0.276, 0.316 and 0.217, respectively, which were significant at the level of *p* < 0.01, indicating that the self-efficacy dimension of psychological capital has a significant positive impact on knowledge mastery, knowledge application and knowledge innovation. Knowledge mastery, knowledge application and knowledge innovation have no significant effect on the three dimensions of learning performance in the hope and optimism dimensions of psychological capital. The regression coefficients of knowledge mastery, knowledge application and knowledge innovation in the resilience dimension of psychological capital were 0.534, 0.422 and 0.417, respectively, which were significant at the level of *p* < 0.01. It can be said that the resilience dimension of psychological capital has a significant positive effect on knowledge mastery, knowledge application and knowledge innovation. From the comparison of the regression coefficients, the regression coefficients of resilience in knowledge mastery, knowledge application and knowledge innovation were greater than those of self-efficacy in knowledge application and knowledge innovation, which shows that resilience has a higher positive impact on knowledge application and knowledge innovation than self-efficacy.

#### 5.3.3. Analysis of the Mediating Effect of Psychological Capital

From the regression analysis of psychological capital’s impact on learning performance, we can see that the self-efficacy dimension of psychological capital has a significant effect on the three dimensions of learning performance, and the resilience dimension has a significant effect on the knowledge application and mastery of learning performance. Therefore, this paper analyzed the intermediary effect of psychological capital from the dimensions of self-efficacy and resilience of psychological capital.

The mediating effect of self-efficacy between competition and learning performance is shown in Table 2. The minimum tolerance of the explanatory variables of the regression model was 0.675, and the maximum VIF was 1.668, which indicates that there is no collinearity among the variables. It can be seen from Table 2 that the F values of the regression model were 20.227, 22.636 and 19.904, which were significant at the level of *p* < 0.01. The regression coefficients of self-efficacy in learning performance in Model 1, Model 2 and Model 3 were 0.456, 0.475 and 0.469, respectively, and were significant at the level of *p* < 0.01. In Model 1, Model 2 and Model 3, the regression coefficient of direct competition in knowledge mastery at the level of 0.1 was 0.103, while the regression coefficients of direct competition in knowledge application and knowledge innovation at the level of 0.05 were 0.126 and 0.125, respectively, which shows that self-efficacy plays an intermediary role between direct competition and learning performance, that is, the influence of direct competition on learning performance is partly realized by self-efficacy. However, in Model 1, Model 2 and Model 3, indirect competition did not pass the significance test for knowledge mastery, knowledge application and knowledge innovation, which shows that there is no intermediary transmission path effect between indirect competition and learning performance.

The mediating effect of resilience between competition and learning performance is shown in Table 3. The minimum tolerance of the explanatory variables of the model was 0.642, and the maximum VIF was 1.710, which indicates that there is no collinearity among the variables. It can be seen from Table 3 that the F values of the regression models of Model 4, Model 5 and Model 6 were 26.609, 25.435 and 24.859, respectively, which were significant at the level of *p* < 0.01. In Model 4, Model 5 and Model 6, the regression coefficients of resilience in knowledge mastery, knowledge application and knowledge innovation were 0.581, 0.516 and 0.547, respectively, which were significant at the level of *p* < 0.01. In Model 4, the regression coefficient of direct competition in knowledge mastery at the level of 0.05 was 0.146, while in Model 5 and Model 6, the regression coefficients of direct competition in knowledge application and knowledge mastery at the level of 0.01 were 0.154 and 0.145, respectively, which shows that resilience plays an intermediary role in the relationship between direct competition and knowledge mastery, knowledge application and knowledge innovation, that is, the influence of direct competition on knowledge mastery, knowledge application and knowledge innovation is partly realized through resilience. In Model 4 and Model 6, the regression coefficients of indirect competition in knowledge mastery and knowledge innovation at the level of 0.05 were 0.119 and 0.122, respectively. Model 5 had a regression coefficient of 0.135 at the level of 0.01 for indirect competition, which shows that resilience plays an intermediary role in the relationship between indirect competition and knowledge mastery, knowledge application and knowledge innovation, that is, the influence of indirect competition on knowledge mastery, knowledge application and knowledge innovation is realized through resilience.

### 5.4. Regulatory Effect Test

The minimum tolerance of the explanatory variables and the maximum VIF were 0.675 and 1.680, respectively, indicating that there is no collinearity between the variables. The adjustment effect of connected classroom atmosphere is shown in Table 4.

The F value of Model 7 was 8.049, which was significant at the level of 0.01, which shows the effectiveness of the model. The regression coefficient of direct competition * classroom atmosphere was 0.146, which was significant at the level of 0.05, which shows that connected classroom atmosphere has a moderating effect on the relationship between direct competition and knowledge mastery. The F value of Model 8 was 8.774, which was significant at the level of 0.01, which shows the validity of the model. The regression coefficient of indirect competition * classroom atmosphere was 0.158, which was significant at the level of 0.05, showing that connected classroom atmosphere has a moderating effect on the relationship between indirect competition and knowledge mastery. The F value of Model 9 was 12.010, which was significant at the level of 0.01, indicating the effectiveness of the model. The regression coefficient of direct competition * classroom atmosphere was 0.051, which failed to pass the significance test, indicating that connected classroom atmosphere has no moderating effect on the relationship between direct competition and knowledge application. The F value of Model 10 was 12.323, which was significant at the level of 0.01, indicating the effectiveness of the model. The regression coefficient of indirect competition * classroom atmosphere was 0.060, which failed to pass the significance test, indicating that the moderating effect of connected classroom atmosphere on the relationship between indirect competition and knowledge application is not significant. The F value of Model 11 was 12.055, which was significant at the level of 0.01, which shows the effectiveness of the model. The regression coefficient of direct competition * classroom atmosphere was 0.073, which was significant at the level of 0.1, which shows that connected classroom atmosphere has a significant moderating effect on the relationship between direct competition and knowledge innovation. The better the connected classroom atmosphere, the greater the influence of direct competition on knowledge innovation. The F value of Model 12 was 11.064, which was significant at the level of 0.01, which shows the effectiveness of the model. The regression coefficient of indirect competition * classroom atmosphere was significant at the level of 0.05, which shows that connected classroom atmosphere has a moderating effect on the relationship between indirect competition and knowledge innovation, which shows that the better the connected classroom atmosphere, the stronger the positive impact of indirect competition on knowledge innovation.

### 5.5. Discussion and Analysis

First, online learning performance is the lifeline of online education, and it is also the core indicator of online education quality evaluation and popularization. There are many ways to present the application of game competition in interactive learning. Scholars have investigated and analyzed the influence of specific game competition modes on the learning effect and drawn meaningful conclusions. For example, foreign scholars have found that the introduction of ranking competition will reduce the final exam scores of mathematics courses; friendship competition games will help improve learning performance; and serious game competition will improve learners’ learning motivation and academic performance. There is work by foreign scholars on the online learning interactive links of the game-based competitive design of the learning results of the study of the effect of online learning under the indirect competitive relationship. Chen Guoqing, a domestic scholar, put forward the idea of classifying game competition for the first time. From the analysis of behavioral data of a learning platform, it was found that indirect competitive interaction represented by ranking and direct competitive interaction represented by competition can improve the number of words memorized every week. Based on the existing research, this study analyzed the game competition relationship in the interactive module of online learning. This paper classifies and defines the game-based competition modes and draws a conclusion through empirical research that both direct competition and indirect competition have significant effects on the three dimensions of knowledge mastery, knowledge application and knowledge innovation of online learning performance, which enriches the research system of game-based competition and online learning.

Secondly, this study puts forward the mediating role of psychological capital between game competition and learning performance for the first time. The empirical analysis showed that the influence of hope and optimism on learning performance did not pass the significance test. The reason may be that the samples have differences in psychological capital as a whole, but there is no obvious difference in the dimensions of hope and optimism. It may also be because of the special attribute of the online learning situation. The learning process is a type of autonomous learning process in virtual situations. On the one hand, learners have the basic cognition of learning results attributable to the learning subject; on the other hand, MOOC learning itself is a self-charging process, which requires learners to work hard, so the variables of the emotional dimension of psychological capital cannot produce significant differences in learning performance. The self-efficacy dimension of mental capital plays an intermediary role between direct competition and learning performance, while the resilience dimension of mental capital plays an intermediary role between direct competition and learning performance, and between indirect competition and learning performance. This study is the first to propose and validate the mediating role of psychological capital between game-based competition and learning performance.

Finally, a connected classroom atmosphere significantly regulates the relationship between competition and knowledge mastery, and between competition and knowledge innovation, but it has no significant regulating effect on the relationship between competition and knowledge application. The idea behind knowledge application is to examine learners’ internalization and absorption of learned knowledge, participate in the activities of applying what they have learned and test it in practice, so as to improve their ability to apply knowledge. Competitive learning activities stimulate learners’ inner psychological environment, stimulate or inhibit learners’ learning motivation, enthusiasm and sense of accomplishment, form learners’ psychological space and adjust learners’ learning effect. A harmonious and cheerful classroom atmosphere is conducive to learners’ learning experience. However, online MOOC learning mainly focuses on learning theoretical knowledge or basic knowledge of courses, and practical application has always been the weak link of online learning. A connected classroom atmosphere is only an environmental factor, which cannot regulate competition and knowledge application in the virtual online learning situation.

## 6. Conclusions and Enlightenment

This study was based on online learning scenarios, field theory, psychological capital and constructivist learning theory and explored the mechanism of game-based competition’s impact on learning performance through quantitative research. The results show that direct competition and indirect competition have a significant impact on the three dimensions of online learning performance: knowledge mastery, knowledge application and knowledge innovation. This shows that the application of game-based competition in the interactive module of online learning is beneficial to the improvement of learners’ learning performance. At the same time, the self-efficacy dimension of mental capital plays an intermediary role between direct competition and learning performance. The resilience dimension of mental capital plays an intermediary role between the two dimensions of competition and the three dimensions of learning performance. Connected classroom atmosphere plays a regulating role in the dimensions of direct competition, knowledge mastery and knowledge innovation, as well as a moderating role in indirect competition, knowledge mastery and knowledge innovation. This shows that learners with high self-efficacy and resilience will set higher and higher goals for themselves, challenge themselves, mobilize resources, overcome difficulties, motivate themselves and achieve the established goals. A friendly, relaxed and pleasant classroom atmosphere can adjust anxiety and other related negative emotions caused by competitive pressure in the learning process, thus improving online learning performance.

The innovations of this study are as follows: Firstly, this study defines the game-based competition relationship in the interactive module of online learning, classifies the game-based competition modes and demonstrates that both direct competition and indirect competition have positive effects on online learning performance. Secondly, the mediating variable psychological capital is introduced to adjust the effect of game competition and learning performance, and the mediating effect of the self-efficacy and resilience dimensions of psychological capital is found. Thirdly, the moderating effect of connected classroom atmosphere on the knowledge mastery and knowledge innovation dimensions of learning performance is found.

The conclusion of this study provides enlightenment for the design of online curriculum resources and curriculum platform enterprises. First of all, in the construction of online resources, game-based competition can be designed to stimulate learners’ interest in learning, improve the stickiness of the curriculum platform and improve online learning performance. At the same time, we should pay attention to the matching degree between learning content and learners’ knowledge reserve in game-based competitive learning and set up a competitive mode of “jumping and reaching” to stimulate learners’ desire to challenge, and motivation to repeat learning. Secondly, hierarchical learning activities can be carried out using the learning behavior data of learners and the characteristic attributes of individual learners on the curriculum platform. For learning with high self-efficacy, good resilience and strong resilience, this can improve the learning difficulty, broaden the knowledge scope, incorporate innovative elements and promote learners’ deep learning. Thirdly, in online learning competition and interaction, we should pay attention to creating a good interactive atmosphere, reducing anxiety and psychological tension caused by competitive pressure and enabling learners to have comfortable and pleasant learning experiences and to obtain a learning effect. Finally, for the curriculum platform enterprises, they can develop online learning modules of multi-type game interaction and push effective learning interaction methods according to learners’ psychological capital characteristics and learning goals.

## Figures and Tables

**Table 1:** Correlation regression analysis between competition and learning performance.

Variable	KM	KA	IA
Control variables			
Gender	−0.006 (−0.061)	0.031 (0.319)	−0.042 (−0.420)
Place of origin	−0.001 (−0.027)	−0.069 (−1.296)	−0.113 (−1.538)
Education level	0.211 (1.235)	0.216 (1.213)	0.142 (0.775)
Grade	0.030 (0.620)	0.068 (1.331)	0.123 (1.577)
Degree category	−0.123 (−1.508)	−0.075 (−1.511)	−0.077 (−1.565)
MOOC learning experience	−0.009 (−0.169)	0.086 (1.492)	0.063 (1.062)
Self-varying variables			
Direct competition	0.237 *** (4.124)	0.264 *** (4.421)	0.262 *** (4.269)
Indirect competition	0.194 *** (3.372)	0.201 *** (3.360)	0.158 ** (2.570)
F	10.775 **	12.911 ***	11.403 ***
∆R2	0.227	0.264	0.238

Note: ** *p* < 0.05; *** *p* < 0.01.

**Table 2:** Mediation effect test of self-efficacy.

Variable	KM	KA	IA
	Model 1	Model 2	Model 3
Control variables			
Gender	0.076 (0.918)	0.117 (1.344)	0.043 (0.473)
Place of origin	0.053 (1.163)	−0.012 (−0.256)	−0.057 (−1.142)
Education level	0.165 (1.089)	0.168 (1.063)	0.094 (0.575)
Grade	0.045 (1.042)	0.083 (1.544)	0.138 (1.560)
Degree category	−0.128 * (−1.987)	−0.080 (−1.561)	−0.082 (−1.514)
MOOC learning experience	−0.027 (−0.547)	0.068 (1.328)	0.045 (0.847)
Self-varying variables			
Direct competition	0.103 * (1.948)	0.126 ** (2.268)	0.125 ** (2.178)
Indirect competition	0.048 (0.894)	0.049 (0.883)	0.008 (0.140)
Mediating variable			
Self-efficacy	0.456 *** (8.491)	0.475 *** (8.486)	0.469 *** (8.075)
F	20.227 ***	22.636 ***	19.904 ***
∆R2	0.394	0.423	0.390

Note: * *p* < 0.1; ** *p* < 0.05; *** *p* < 0.01.

**Table 3:** Mediating effect of toughness.

Variable	KM	KA	IA
	Model 4	Model 5	Model 6
Control variables			
Gender	−0.062 (−1.351)	−0.065 (−0.763)	−0.143 (−1.501)
Place of origin	0.010 (0.226)	−0.059 (−1.274)	−0.102 (−1.538)
Education level	0.098 (1.355)	0.263 (1.527)	0.192 (1.228)
Grade	0.045 (0.935)	0.075 (1.514)	0.131 (1.563)
Degree category	−0.120 * (−1.954)	−0.071 (−1.521)	−0.073 (1.391)
MOOC learning experience	−0.007 (−0.154)	0.088 * (1.774)	0.065 (1.291)
Self-varying variables			
Direct competition	0.146 ** (2.462)	0.154 *** (2.906)	0.145 *** (2.698)
Indirect competition	0.119 ** (2.230)	0.135 *** (2.560)	0.122 ** (2.464)
Mediating variable			
Toughness	0.581 *** (8.491)	0.516 *** (9.487)	0.547 *** (9.907)
F	26.609 ***	25.435 ***	24.859 ***
∆R2	0.464	0.453	0.447

Note: * *p* < 0.1; ** *p* < 0.05; *** *p* < 0.01.

**Table 4:** Adjusting effect of connected classroom atmosphere.

Variable	KM		KA		IA	
	Model 7	Model 8	Model 9	Model 10	Model 11	Model 12
Control variables						
Gender	−0.053 (−0.669)	−0.045 (−0.570)	−0.009 (−0.109)	−0.006 (−0.070)	−0.085 (−0.939)	−0.072 (−0.797)
Place of origin	0.029 (0.670)	0.027 (0.630)	−0.040 (−0.868)	−0.042 (−0.900)	−0.087 (−1.441)	−0.089 (−1.490)
Education level	0.203 (1.409)	0.219 (1.517)	0.210 (1.357)	0.225 (1.448)	0.134 (0.813)	0.153 (0.926)
Grade	0.065 (1.565)	0.061 (1.491)	0.103 (1.552)	0.098 (1.509)	0.152 (1.499)	0.150 (1.508)
Degree category	−0.102 (1.569)	−1.02 (−1.501)	−0.059 (−1.539)	−0.055 (−1.458)	−0.056 (−1.387)	−0.061 (−1.496)
MOOC learning experience	−0.020 (0.664)	−0.021 (−0.155)	0.076 (1.530)	0.074 (1.492)	0.053 (0.990)	0.053 (0.990)
Self-varying variables						
Direct competition	−0.052(−0.333)	0.132 *** (2.667)	−0.129 (−0.769)	0.164 *** (3.089)	0.065 (0.363)	0.171 (3.306)
Indirect competition	0.182 ** (3.195)	−0.150 (−0.985)	0.092 ** (2.108)	−0.143 (−0.859)	0.396 *** (3.324)	−0.195 ** (−1.117)
Regulating variable						
Classroom atmosphere	0.319 *** (3.403)	0.356 *** (3.557)	0.055 (0.951)	0.080 (1.494)	0.332 *** (2.983)	0.275 ** (2.010)
Product terms						
DC *CA	0.146 ** (1.919)		0.051 (0.912)		0.073 * (1.841)	
IC *CA		0.158 ** (1.934)		0.060 (1.458)		0.165 ** (1.945)
F	8.049 ***	8.774 ***	12.010 ***	12.323 ***	12.055 ***	11.064 ***
∆R2	0.205	0.213	0.220	0.245	0.241	0.232

Note: * *p* < 0.1; ** *p* < 0.05; *** *p* < 0.01.

## Data Availability

Not applicable.
